# Perspectives of researchers, science policy makers and research ethics committee members on the feedback of individual genetic research findings in African genomics research

**DOI:** 10.1186/s12910-024-01068-2

**Published:** 2024-06-07

**Authors:** Faith Musvipwa, Ambroise Wonkam, Benjamin Berkman, Jantina de Vries

**Affiliations:** 1 Department of Medicine, University of Cape Town, Cape Town, South Africa; 2 Johns Hopkins Department of Genetic Medicine, Baltimore, USA; 3 NIH Department of Bioethics, Department of Bioethics Bethesda, Bethesda, USA; 4 Ethics Lab Neuroscience Institute, University of Cape Town, Cape Town, South Africa

**Keywords:** Individual genetic research results, Feedback, Genomics research, Incidental findings, Africa

## Abstract

**Background:**

Genetic research can yield information that is unrelated to the study’s objectives but may be of clinical or personal interest to study participants. There is an emerging but controversial responsibility to return some genetic research results, however there is little evidence available about the views of genomic researchers and others on the African continent.

**Methods:**

We conducted a continental survey to solicit perspectives of researchers, science policy makers and research ethics committee members on the feedback of individual genetic research findings in African genomics research.

**Results:**

A total of 110 persons participated in the survey with 51 complete and 59 incomplete surveys received. Data was summarised using descriptive analysis. Overall, our respondents believed that individual genetic research results that are clinically actionable should be returned to study participants apparently because participants have a right to know things about their health, and it might also be a means for research participation to be recognized. Nonetheless, there is a need for development of precise guidance on how to return individual genetic research findings in African genomics research.

**Discussion:**

Participants should receive information that could promote a healthier lifestyle; only clinically actionable findings should be returned, and participants should receive all important information that is directly relevant to their health. Nevertheless, detailed guidelines should inform what ought to be returned. H3Africa guidelines stipulate that it is generally considered good practice for researchers to feedback general study results, but there is no consensus about whether individual genomic study results should also be fed back. The decision on what individual results to feedback, if any, is very challenging and the specific context is important to make an appropriate determination.

**Supplementary Information:**

The online version contains supplementary material available at 10.1186/s12910-024-01068-2.

## Introduction

One of the primary ethical questions in genomics research relates to the return – or not – of individual genetic research results. On-going debate for over a decade in the genetic research sphere reveals a growing consensus that researchers should at the very least offer to return high-impact, medically significant, actionable findings when doing so is not unduly burdensome [1]. There is a growing evidence base that participants across the world – and in Africa – wish to receive such results [2]. On the African continent, an obligation to return individual genetic research results has been grounded in African philosophy [3]. A failure to return results has been said to be increasingly frustrating to study participants, with a detrimental impact on community interest in participating in research [4]. Evidence from Botswana suggests that participants are strongly in favour of returning individual findings, at least partly because this is seen as a key way to respect reciprocity and because it could be of benefit to participants [5]. Furthermore, there is some evidence that suggests that ethics committee members and policy makers in Botswana also support return of results, at least to the extent that it respects participant autonomy [6]. Partly in response to this evolving evidence base, the current Human Heredity and Health in Africa (H3Africa) feedback of findings policy [7] urges researchers to be specific about whether and which results they will return, although it does not require researchers to return results. However, against these developments researchers from Uganda have cautioned that the return of individual genetic findings could have adverse consequences for individuals and their families as well as the entire communities if not handled properly [8]. Furthermore, Wonkam and De Vries have outlined a number of key challenges relating to the return of individual genetic research findings [9]. Some of these challenges are that African populations are far more genetically diverse than non-African populations, which helps to explain why the continent is currently the focus of genomic research. Genetic diversity presents a challenge for sharing individual research results due to its complexity and intricacy. When populations are genetically diverse, it means that there are numerous variations and differences in the genetic makeup of individuals within that population. This diversity can encompass a wide range of genetic traits, including both common and rare variants. The data show that there are fewer pathogenic variants identified in people of African descent in the current literature. This is most likely due to the underrepresentation of participants with African ancestry in clinical and research reports and exome databases, as well as the lack of uniform reporting guidelines for pathogenic variants in African genomic research [9]. To further illuminate these challenges, and to get a broad sense of the views of professionals involved in genomics research on the African continent, we conducted a continental survey with researchers, science policy makers and research ethics committee members.

## Materials and methods

### Methods

We conducted a continent-wide, online survey in English to better understand what researchers, science policy makers and research ethics committee members in Africa think about the return of individual genetic research results.

### Survey development

To develop the survey, we first conducted an in-depth review of the literature on returning research results, including papers specifically from the African continent, to identify key themes and areas of controversy that needed further investigation. We also collected other published survey instruments used to probe views and attitudes from professionals on the return of individual genetic research results [10]. We then identified the following categories that we wanted to probe issues around knowledge on genomics; views on feedback of individual genetic research findings; what findings ought to be returned consent; actionability of genetic research; standard of care that should be applied; reproductive decision making; constraints in returning incidental findings; importance of feeding back findings; cost of feeding back findings and experience of returning individual genetic research results. The survey used in this study was developed for this study only and has not been published elsewhere.

The draft survey was reviewed by five colleagues working in genomics research and revisions were made following their suggestions. An online survey was designed using University of Cape Town (UCT) REDCap. The survey was anonymous; neither the hosting site nor we could trace participants’ IP addresses or associate e-mail addresses with responses.

The self-administered survey consisted primarily of multiple-choice questions, with opportunity for short open-ended text answers (see appendix 1).

### Survey sampling

We circulated the survey link to professional and personal networks on the African continent. Specifically, we circulated the survey link to the broad H3Africa community, which includes genomic researchers, policy makers, ethicists as well as other persons interested in genomics. We also circulated the survey link to continental ethics platforms such as the Global Health Ethics Network, H3Africa and the African Society for Human Genetics. Finally, we circulated the link to persons in our individual networks with a request to further circulate the link to their ethics committees or other relevant participants. Two follow-up e-mails were sent to encourage participation in the survey and thank those who had already participated.

### Data analysis

Survey data was exported from REDCap and responses loaded into Statistical Package for the Social Sciences 27 (SPSS) for descriptive analysis such as frequencies. Survey data were exported from REDCap, and survey responses were analyzed with descriptive statistics (percentages for discrete responses, means and standard deviations for continuous responses). Frequency statistics were analysed to produce summary measures for categorical variables in the form of frequency tables, bar charts, or pie charts.

Descriptive statistics are procedures that describe numerical data in that they assist in organising, summarising and interpreting sample data. We used frequency analysis, which is a descriptive statistical method that shows the number of occurrences of each response chosen by the respondents then draw conclusions. After having collected the data, it was prepared for data entry. Data was checked, edited, and coded into a format that is machine-readable. After designing a questionnaire, selecting a sample, and collecting data a code sheet was created. Code sheets are useful in that they provide both the guide and the record of how the responses gathered from the questionnaire are to be coded. An important point to note, with regards to the code sheet, is that every response should be coded, including non-responses. This is so that all responses to every question can be accounted for, both in the analysis of the data and importantly when checking for data entry errors [11, 12].

### Ethical considerations

Participants were informed that they would be presumed to have consented to the survey if they initiated the survey by clicking ‘‘Next’’ on the first page which provided information about the purpose, risks, and benefits of the study. No compensation was provided. This study was approved by the Human Research Ethics Committee of the UCT Faculty of Health Sciences as part of the IFGENERA study (FHS HREC 782–2018), the study did not receive its own independent approval as it was covered by the parent study. IFGENERA is part of the Human Heredity and Health in Africa (H3Africa) Consortium.

## Results

The results are illustrated below in the form of numbers and percentages.

### Demographic characteristics of study population

Table 1 below summarizes the demographic characteristics of research participants. It presents their highest level of education, role in research, research experience, years served on ethics committee and years of experience in science policy development.

**Table 1:** Demographic characteristics of study population

Description	Item	Number (*n*) and percentage (%)
Highest level of education	Doctoral	(*n* = 43, 47%)
		(*n* = 37, 42%)
	BSc	(*n* = 9, 10%)
	Other	(*n* = 2, 2%)
Role in research	Researcher	(*n* = 60, 68%)
	Team leader	(*n* = 17, 19%)
	Ethics committee member	(*n* = 23, 26%)
	Science policy maker	(*n* = 3, 3%)
	Other	(*n* = 7, 8%)
Research experience	Less than 1 year	(*n* = 6, 9%)
	1–2 years	(*n* = 15, 22%)
	3–5 years	(*n* = 20, 29%)
	6–10 years	(*n* = 11, 16%)
	More than 10 years	(*n* = 16, 24%)
Years served as ethics committee member	Less than 1 year	(*n* = 3, 13%)
	1–2 years	(*n* = 2, 9%)
	3–5 years	(*n* = 11, 49%)
	6–10 years	(*n* = 1, 4%)
	More than 10 years	(*n* = 6, 26%)
Years of experience in science policy development	6–10 years	(*n* = 1, 33%)
	More than 10 years	(*n* = 2, 67%)

A total of 110 persons participated in the survey, of which 51 were complete survey responses and 59 were incomplete responses. All responses were analysed and included hence differences on number of responses on each question. Most survey respondents were well-educated with about half of participants having obtained doctoral degrees 43 (47%), while 37(42%) had MSc degrees, whereas 9 (10%) had BSc degrees and 19 (17%) chose not to respond. The largest number of responses came from researchers and research team leaders, who together made up almost 90% of respondents 77 (87%). About a quarter 23 (26%) of respondents served on an ethics committee while 17 (19%) were team leaders and 7 (8%) indicated other. Of the researchers, about a third of respondents had between 3 and 5 years of experience, and a quarter of respondents had more than 10 years’ experience. Half of the 23 respondents who were ethics committee members, had between 3 and 5 years experiences whilst a quarter had more than 10 years’ experience in serving on the ethics committee. On the years of experience in science policy development, 2 (67%) had more than 10 years’ experience whilst one person had 6–10 years’ experience.

### Knowledge about genomics

Table 2 below depicts respondents’ level of understanding ethical issues in genomic research, familiarity with issues around return of individual genetic results in Africa, and how familiar they are with the H3Africa guideline for return of genetic research findings.

**Table 2:** Knowledge about genomics

How confident are you in your understanding of the ethical issues (such as privacy, confidentiality, informed consent and return of individual results) that might arise from genomic research?	Number/percentage
Very confident	(*n* = 48, 57%)
Somewhat confident	(*n* = 27, 32%)
Slightly confident	(*n* = 7, 8%)
Not at all confident	(*n* = 2, 3%)
**How familiar are you with issues around the return of individual genetic research results in African genomics (e.g., have you heard about individual findings in training, have you fed back results in the context of genomics research, have you read about individual findings in literature etc.)**	
Very confident	(*n* = 26, 32%)
Somewhat confident	(*n* = 30, 37%)
Slightly confident	(*n* = 11, 14%)
Not at all confident	(*n* = 14, 17%)
**How familiar are you with the H3Africa Guideline for the Return of Individual Genetic Research Findings?**	
Very familiar	(*n* = 15, 18%)
Somewhat familiar	(*n* = 28, 34%)
Slightly familiar	(*n* = 15, 18%)
Not at all familiar	(*n* = 24, 30%)

Overall, half of the respondents 48 (57%) indicated that they were very confident in their understanding of ethical issues (such as privacy, confidentiality, informed consent and return of individual results) that might arise from genomic research. A total of 27 respondents (32%) indicated that they were somewhat confident, while 7 respondents (8%) were slightly confident and only 2 (2%) were not at all confident. Most of the respondents indicated that they were either very 26 (32%) or somewhat 30 (37%) confident in being familiar with issues around the return of individual genetic research results in African genomics: they had heard about individual findings in training, had experience with feeding back of results in the context of genomics research or read about feedback of individual findings in literature. The rest was only slightly 11 (14%) or not at all 14 (17%) confident in these issues. Overall, our respondents seemed knowledgeable of and confident in their understanding of ethical issues in genomics research in general, and issues around the return of individual genetic research results more specifically.

The H3Africa guidelines for the return of individual genetic research results [7] lays out several crucial criteria to help H3Africa researchers decide whether to return individual genetic research findings to research participants. The guidelines include a flowchart, which provides a logical framework for planning and deciding whether to offer individual feedback. Highest number of the respondents 28 (34%) were somewhat familiar, and 15 (18%) familiar, while 28 (34%) were somewhat familiar or slightly 15 (18%) familiar with the H3Africa guidelines for return of results, while 24 (29%) were not at all familiar with these guidelines.

### Views on feedback of individual genetic research findings

**Fig. 1:**
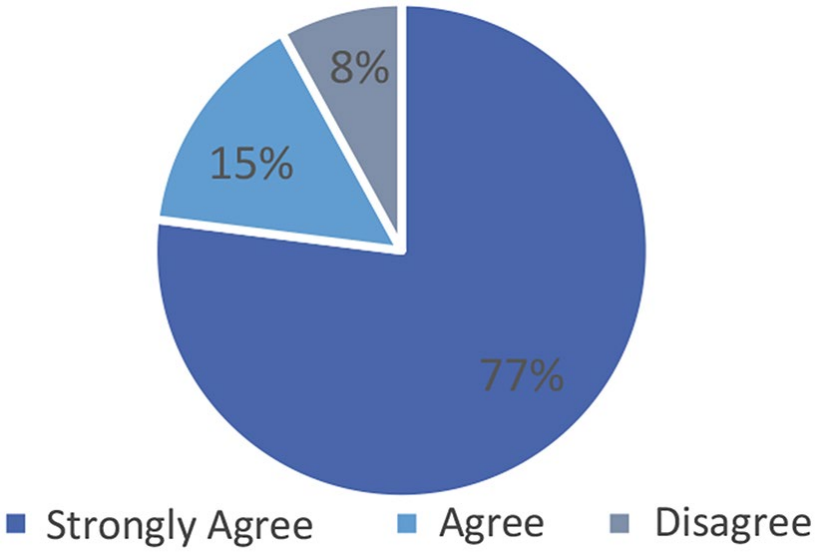
Returning individual genetic results

Figure 1 below shows respondents’ level of agreement to returning individual genetic research results. A total of 24 participants (92%) either strongly agreed or agreed that individual genetic results should be returned and 2 (8%) disagreeing. None of the participants strongly disagreed or had neutral views on returning individual genetic results while 84 did not answer this question, suggesting that most of our respondents were unsure about this question. In response to an open-ended question to share with us any relevant information about their experience on return of findings and responses thematically analysed, a common theme was that returning findings should be culture-sensitive, especially in African settings. Respondents also mentioned that not returning results may have a negative effect on the participation in new projects and could affect the participants’ trust in the researchers and that researchers have a moral obligation “to help” participants who had helped them in their studies through participation, by not only feeding back individual findings but also providing care whenever possible.

In response to close-ended questions when asked about the reasons for feedback, a high number 41 (77%) of the respondents noted that the most important reason for feedback of individual genetic research results is to appreciate participants’ contribution to research (See Table 6). An almost equal number 40 (76%) indicated that return of research results would be one way in which research participation could be reciprocated. A total of 33 respondents (62%) indicated that receiving individual genetic research results would show participants that their participation was valued, and 22 (42%) respondents thought that participants should receive their individual genetic research results in exchange for their participation in research.

### Characteristics of views about whether genetic results should be returned

Table 3 below illustrates respondents’ views on which genetic research findings should be returned. Respondents had an option to select all that apply. Most of the respondents 49 (64%) indicated that there should be clear guidance about how to go about the return of individual genetic research findings in African genomics. A total of 44 (58%) respondents indicated that participants have a right to know things about their health that are revealed in African genomics, whereas 40 (53%) indicated that returning some findings is an important way of giving back to the people who participated in the research. However, 38 (50%) of the respondents were of the view that decisions about what should be returned should be made on a case-by-case basis, and they should be made in consultation with the ethics committee. Only a minority of respondents 2 (3%) indicated that individual genetic research results should never be returned in African genomics because there are too many questions and uncertainties.

**Table 3:** Views on returning individual ggenetic results

Which of the following best characterizes your view on return of individual genetic research results? Please tick all that apply	
Individual genetic research results should never be fed back in African genomics; there are too many questions and uncertainties	(*n* = 2, 3%)
Returning some findings is an important way of giving back to the people who participated in the research (reciprocity)	(*n* = 40, 53%)
Participants have a right to know things about their health that are revealed in African genomics	(*n* = 44, 58%)
Decisions about what should be returned should be made on a case-by-case basis, and they should be made in consultation with the ethics committee	(*n* = 38, 50%)
There should be clear guidance about how to go about the return of individual genetic research findings in African genomics	(*n* = 49, 64%)
Other	(*n* = 2, 3%)

*Data are number (percentage) of respondents. Because the question required a tick all that applies, percentages do not add up to 100.

Table 4 below describes close ended responses on what is important to respondents when deciding which information should be returned to genetic research participants. Most respondents 43 (70%) indicated that participants should receive information that could promote a healthier lifestyle and motivate changes in lifestyle. 37 respondents (60%) suggested that participants should only receive information about their health if that information is clinically actionable and could prevent severe disease, whereas 34 (55%) were of the view that participants should receive all information that is directly relevant to their health. A total of 23 (37%) respondents were of the view that participants should have access to as much information as possible and 22 (36%) indicated that participants should receive all information about their health even if that information is not clinically actionable.

**Table 4:** **Decision on what information to feedback**

When deciding on what information to feedback, what do you think is most important? Please tick all that apply	Number (percentage)
Participants should have access to as much information as possible.	(*n* = 23, 37%)
Participants should receive all information that is directly relevant to their health.	(*n* = 34, 55%)
Participants should only receive information about their health if that information is clinically actionable (“Clinically actionable” means that there is effective prevention or treatment available through medical care) and could prevent severe disease.	(*n* = 37, 60%)
Participants should receive all information about their health even if that information is not clinically actionable.	(*n* = 22, 36%)
Participants should receive information that could promote a healthier lifestyle and motivate changes in lifestyle.	(*n* = 43, 70%)

*Data are number (percentage) of respondents. Because the question required a tick all that applies, percentages may not add up to 100 they are more.

### Views on returning genetic results to paediatric patients

**Fig. 2:**
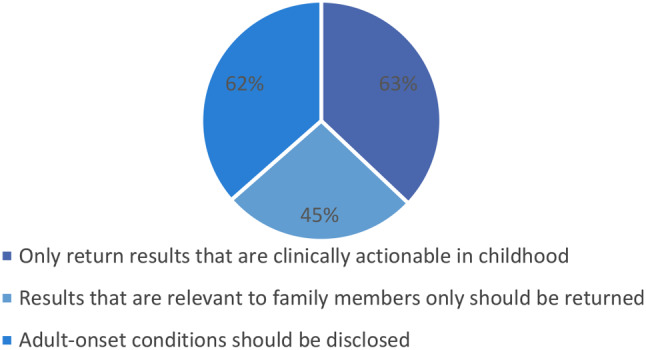
Views on returning genetic results to paediatric patients. *Data are number (percentage) of respondents. Because the question required a tick all that applies, percentages may not add up to 100 they are more.

Figure 2 below represents respondents’ views on which genetic findings should be returned to paediatric patients. Respondents had an option to tick all that apply. A majority 39 (63%) of respondents indicated that only results that are clinically actionable in childhood should be returned (where “clinically actionable” means that there is effective prevention or treatment available through medical care), while 38 respondents (62%) were of the view that adult-onset conditions should also be disclosed (where “adult-onset conditions” means conditions that may manifest themselves after childhood and adolescence) and 28 (45%) were of the view that results that are relevant to family members only should be returned.

### Kinds of genetic results to be feedback

**Fig. 3:**
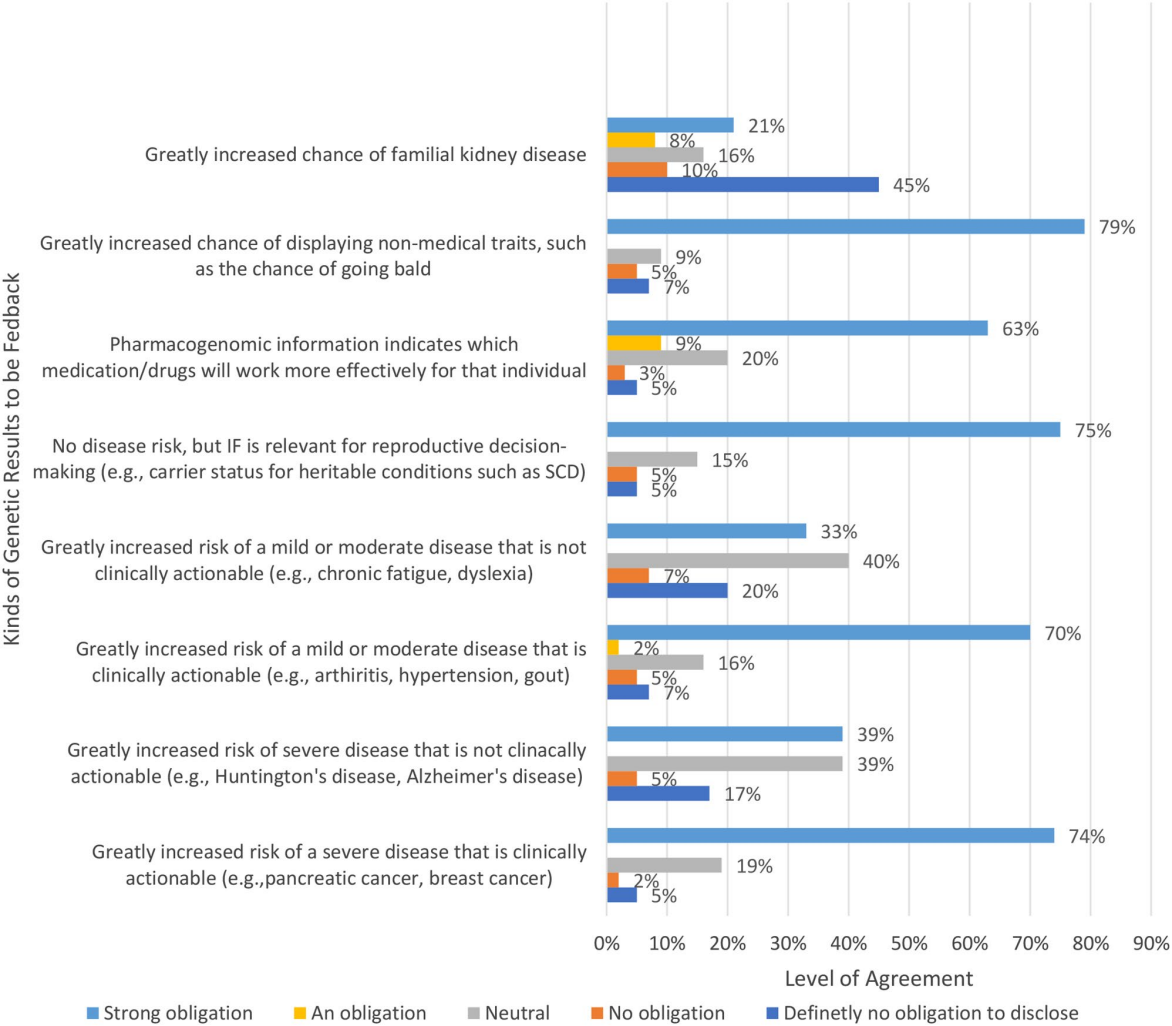
Kinds of genetic results to be feedback

Figure 3 below illustrates respondents’ views on which kinds of genetic research findings should be returned. Responses were provided using a scale from 1 to 5, where 1 represents no obligation to feedback and 5 represents a strong obligation to feedback findings. See Fig. 3. Most of the respondents indicated that there is a strong obligation to feedback findings that are clinically actionable and a strong obligation to feedback if findings are relevant for reproductive decision making.

### Consent

**Fig. 4:**
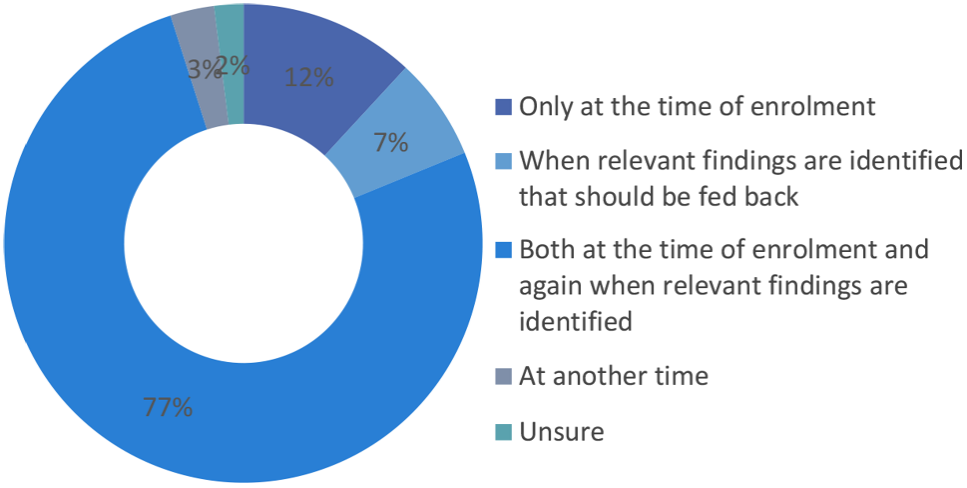
At which point should consent to receive findings be given

Figure 4 below portrays at which point should participants give consent to receive incidental individual genetic research findings. Most respondents 46 (77%) indicated that consent for return of incidental findings should be given both at the time of enrolment and again when relevant findings are identified, while 7 (12%) suggested only at the time of enrolment. A mere 4 respondents (7%) indicated that consent should be given when relevant findings are identified that should be returned.

**Fig. 5:**
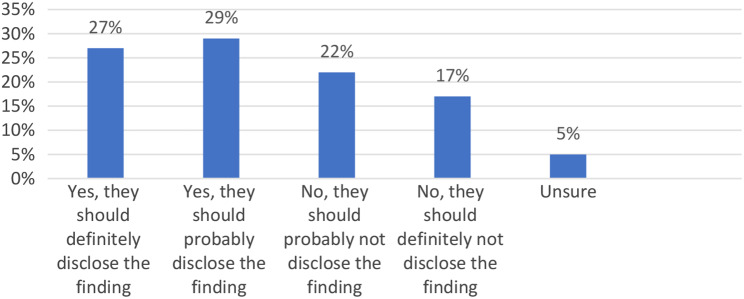
Disclosing results when participant chose not to receive

We also asked respondents what researchers should do if they find something of clinical relevance to an individual who did not consent to receive feedback through a scenario (See Fig. 5). In the scenario, a participant has chosen not to receive any incidental findings (IF) results. During analysis, the research team finds evidence of high genetic risk for a hereditary form of cancer that is unlikely to have been diagnosed. The team believes this information will prevent serious disease and could save the life of the participant. Should the team disclose the finding, even though the participant indicated that he/she did not want to receive any IFs?

A plurality of the respondents 17 (29%) thought that in this case, researchers should probably disclose the finding even if the participant had chosen not to receive them. A total of 16 (27%) indicated that yes, they should definitely disclose the finding, while 13 (22%) indicated, they should probably not disclose the finding and a total of 10 (17%) indicated, they should definitely not disclose the finding. A mere 3 (5%) were unsure.

### Actionable findings and standard of care

**Fig. 6:**
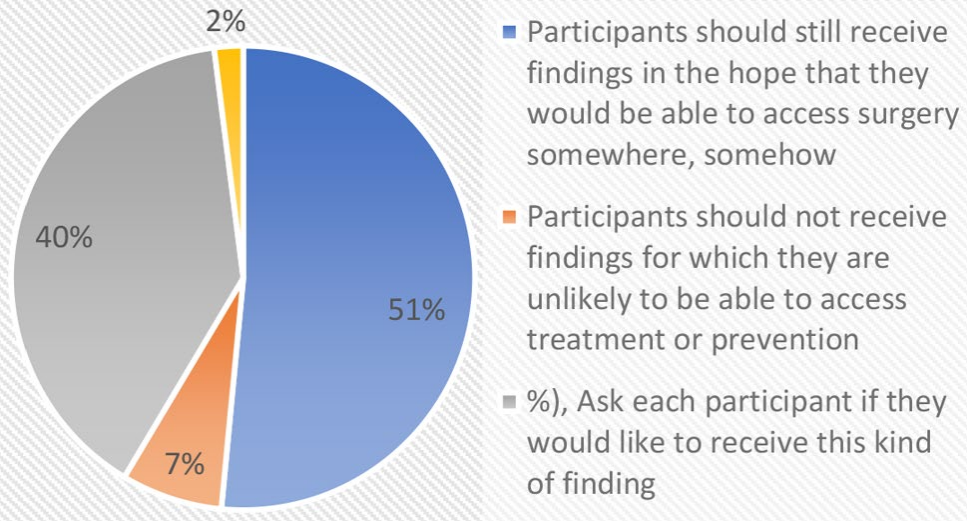
Actionable findings

A recurring issue in African genomics research relates to whether results should be returned to participants when they are not practically actionable because of access/resource limitations [9]. We probed this using a scenario where surgery was indicated but not available in the area where the genomic research was conducted (See Fig. 6). About half 30 (51%) of the respondents indicated that participants should still receive findings in the hope that they would be able to access surgery somewhere, somehow. A total of 23 respondents (40%) suggested asking each participant if they would like to receive this kind of finding whereas 4 (7%) indicated that participants should not receive findings for which they are unlikely to be able to access treatment or prevention.

**Fig. 7:**
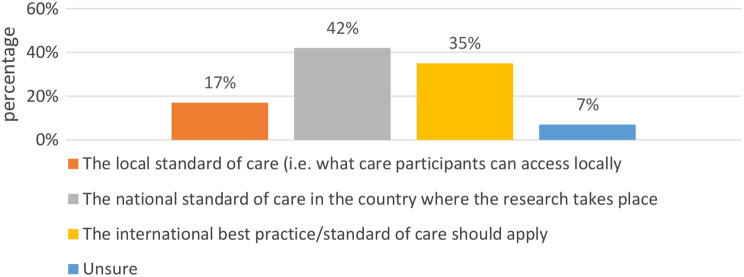
Standard of care

A related question in African genomics is which standard of care should be used to determine whether results are actionable, and whether that should be the national or international standard of care. In many African countries, there is a marked difference in the healthcare that is available in rural vs. urban areas. Using a scenario (See Fig. 7), we probed whether the local, national, or international standard of care should guide decisions to feedback results. Most respondents 25 (42%) indicated that national standard of care in the country where the research takes place should be considered when feeding back individual genetic research results. A total of 21 respondents (35%) suggested that international best practice/standard of care should apply while 10 (17%) suggested that the local standard of care (i.e. what care participants can access locally) should be considered.

**Fig. 8:**
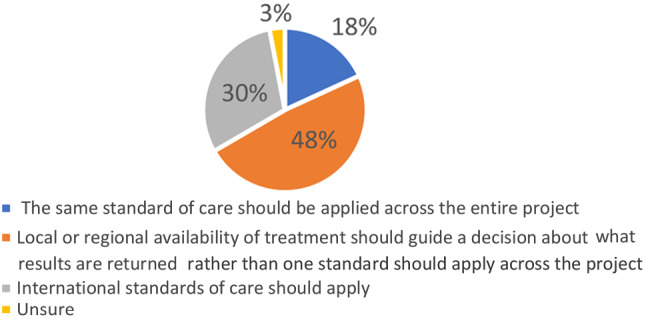
Standard of care across different projects

Another way in which the question of actionability and standard of care comes up in African genomics research is in international collaborative research that takes place across multiple countries that have vastly different standards of care –much as H3Africa research does. The question then is whether the project should adopt one actionability standard that applies to the entire collaboration, or whether the actionability standard should vary for each country in which the research takes place, as has been suggested by some [13]. When asked about this issue (see Fig. 8*)*, the majority 29 (48%) of respondents suggested that national or regional availability of treatment should guide a decision about what results should be returned rather than one standard should apply across the project. A total of 18 respondents (30%) thought that indicated that international standards of care should apply, while 11 (18%) suggested that the same standard of care should be applied across the entire project.

### Reproductive decision making

**Fig. 9:**
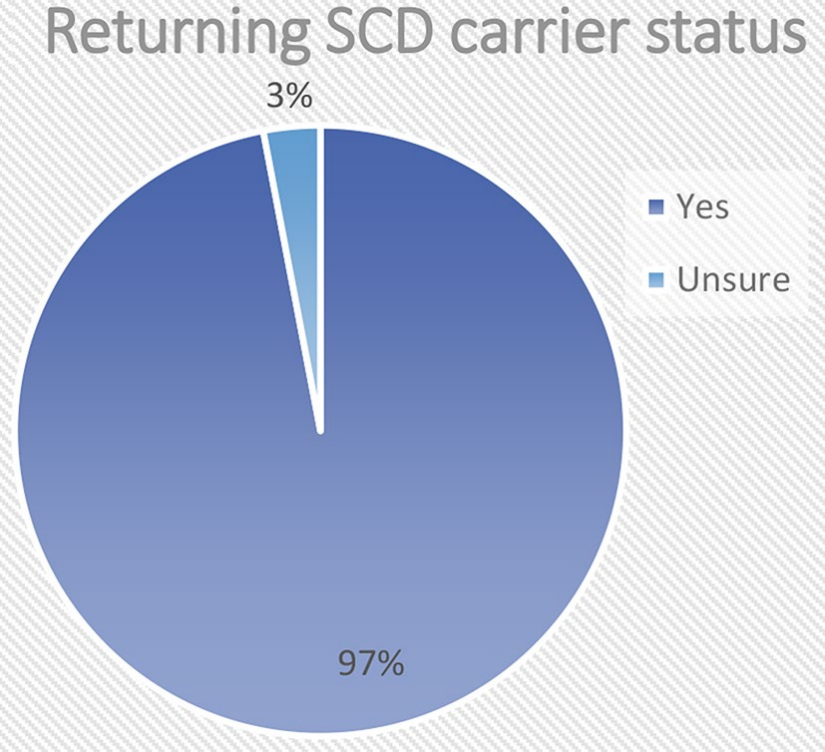
Reproductive decision making

Figure 9 below shows respondent’s views on appropriateness to return information about sickle cell disease (SCD) carrier status in countries with a high burden of SCD. Sickle cell disease (SCD) is a severe hereditary form of anaemia in which a mutated form of haemoglobin distorts the red blood cells into a crescent shape at low oxygen levels. Whilst return of results with reproductive significance are not normally considered for feedback, almost all the respondents in our study who answered this question (*n* = 58, 97%) indicated that it is appropriate to return information about SCD carrier status in countries with a high burden of SCD.

### Challenges to returning incidental findings

Table 5 below shows close-ended responses on what respondent’s views on the most important constraints in returning incidental findings in African genomics research. Respondents could select all that apply. A total of 42 (72%) of the respondents indicated that capacity to interpret the relevance of findings for the health of individuals was the most important constraint in feeding back genetic research findings, while 41 (71%) suggested that absence of genetic diagnostic testing facilities to confirm research findings is the most important constraint in returning incidental findings. However, 40 respondents (69%) indicated that absence of genetic health professionals (trained genetic nurses, genetic counsellors, and medical geneticists) should be considered the most important constraint in returning incidental findings. A total of 37 (64%) suggested that capacity to analyse large datasets for incidental findings is a more important constraint, while 35 (60%) noted difficulty of establishing pathogenicity of variants due to poor representation of Africans in genetic databases as the most important constraint in returning incidental findings and 27 (47%) suggested cost of returning results should be considered as more important in returning incidental findings and a mere 2 (3%) indicated other. (See Table 6)

**Table 5:** Constraints in returning incidental findings

Constraints in returning incidental findings	Number/percentage
Capacity to analyse large datasets for Incidental Findings	(*n* = 37, 64%)
Capacity to interpret the relevance of findings for the health of individuals	(*n* = 42, 72%)
Absence of genetic health professionals (trained genetic nurses, genetic counsellors, and medical geneticists)	(*n* = 40, 69%)
Difficulty of establishing pathogenicity of variants due to poor representation of Africans in genetic databases	(*n* = 35, 60%)
Cost of returning results	(*n* = 27, 47%)
Absence of genetic diagnostic testing facilities to confirm research findings	(*n* = 41, 71%)
Other	(*n* = 2, 3%)

*Data are number (percentage) of respondents. Because the question required a tick all that applies, percentages may not add up to 100 they are more.

**Table 6:** Importance of feeding back findings

Importance of feeding back finds	Number/percentage	
Return of research results would be one way in which research participation could be reciprocated	(*n* = 40, 76%)	
Feedback of individual genetic research results could be a way of appreciating participants’ contribution to research	(*n* = 41, 77%)	
Participants should receive their individual genetic research results in exchange for their participation in research	(*n* = 22, 42%)	
Receiving individual genetic research results would show participants that their participation was valued	(*n* = 33, 62%)	

### Cost of feeding back findings

**Fig. 10:**
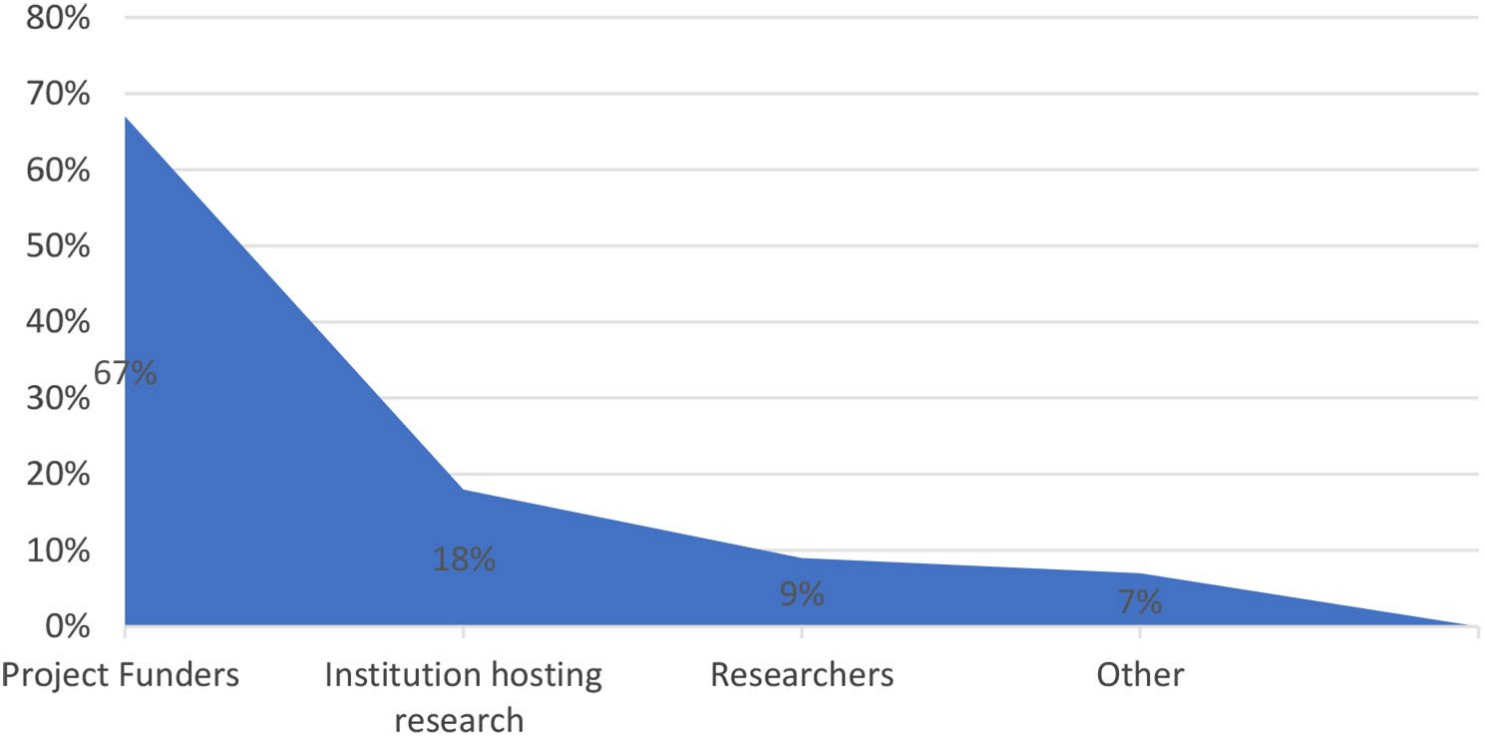
Cost of feeding back findings

Figure 10 below shows respondents on who should incur the costs of feeding back results. The majority 38 (67%) of the participants indicated that project funders should incur the cost of feeding back genetic research findings, while 10 (18%) suggested that the institution hosting the research should incur the cost. A mere 5 (9%) suggested that it should be researchers that incur the costs while 4 (7%) indicated other and none of the respondents suggested that participants should incur the costs.

## Discussion

In this study we surveyed perceptions of African researchers, ethics committee members and science policy makers on the feedback of research results in genomics research in Africa. Amongst our participants there was a strong consensus to return research findings for reasons such as giving back to the community; reciprocity; and to respect participants’ right to know things that concern their health. This poses a challenge on how, when and what findings should be returned. Nevertheless, detailed guidelines should inform what ought to be returned. H3Africa guidelines stipulate that it is generally considered good practice for researchers to feedback general study results, but there is no consensus about whether individual genomic study results should also be fed back. The decision on what individual results to feedback, if any, is very challenging and the specific context is important to make an appropriate determination.

Despite strongly agreeing that research results should be returned, a few were not familiar with the H3Africa guidelines on returning individual research results.

One key finding is the overwhelming support for the return of results of sickle cell disease carrier status. Although there seems to be some support for the return of individual findings of reproductive significance in principle [14], in practice it does not yet seem to be standard practice to return results on carrier status for conditions like Sickle Cell Disease (SCD). Yet in certain parts of Africa, the incidence of SCD is so high that there may be strong ethical arguments to support a policy to return carrier status information to research participants who would not otherwise have access to genetic testing. Our results suggest that such a policy would have strong support in the community of professionals that conduct or govern genomics research on the continent.

Our findings also highlight an underexplored tension in the incidental findings debate, which relates to how actionability should be understood in the context of severe healthcare inequality within and between countries where genomic research is conducted [13]. Overall, we found that there was a division of opinions about whether national or international standards of care should apply in determining actionability, with about a third of our respondents thinking international standards should apply, and a slightly greater proportion thinking national standards should apply. Fewer people were of the view that local standards should apply, or that the same standard should apply across genomic research projects regardless of national standards of care. The spread of opinions on this issue means that it remains unresolved and that more conceptual and – possibly – empirical research is required to resolve this issue in African genomics.

There is some emerging evidence that African research participants expect some individual research results to be returned [4], possibly because of expectations of reciprocity and solidarity [5]. Our respondents – who were not research participants in genomics studies, but people involved in the conduct or governance of genomic research – also considered that reciprocity was a strong reason for the return of individual genetic research results. Providing feedback of findings could be a way in which participants could be recognized for their contribution and could demonstrate researcher solidarity with the community. Furthermore, Tindana and colleagues [4] suggest that the lack of feedback from researchers and the non-return of genetic data are increasingly frustrating to study participants, and this has a possibly detrimental impact on community interest in participating in research. Taken together, our results add further evidence in support of the return of individual genomic research results that are valid, medically important, and actionable.

Our findings suggest that consent to receive findings should be given both at the time of enrolment and again when relevant findings are identified. Any effective method to consent must be interactive and ongoing, only then would it be feasible to deliver information tailored as part of an ongoing conversation. A communicative process that is consent in action and an opportunity to discuss the return of genetic results in advance will be necessary as a model of consent [15].

### Limitations

There are certain limitations to our research that should be considered. Language is one of the study limitations since the survey excluded non-English speakers who conduct genomic research. In addition to the relatively small sample size, our findings could be skewed by non-response bias, since researchers knowledgeable with the topic may have been more eager to take part in the survey. Few policy makers participated in this study which may also have skewed our results. Finally, although 26% of our respondents were ethics committee members, many of those were also researchers which may also have created a further bias in our results.

## Conclusion

Participants should be provided with information aimed at encouraging a healthier lifestyle. Only clinically significant findings should be fed back to them, ensuring that all pertinent information directly related to their health is included. However, it is imperative to establish clear guidelines for determining what should be disclosed. H3Africa guidelines emphasize that it is generally viewed as best practice for researchers to share general study results with participants. Yet, there remains no consensus on whether individual genomic study results should also be shared. The decision regarding which individual results, if any, to share is a complex matter, and the unique context plays a crucial role in making an informed determination.

### Electronic supplementary material

Below is the link to the electronic supplementary material.

Supplementary Material 1

## Data Availability

The datasets used and/or analyzed during the current study are available from the corresponding author on reasonable request.

## Abbreviations

H3Africa: Human Heredity and Health in Africa
UCT: University of Cape Town
REDCap: Research Electronic Data Capture
FHS HREC: Human Research Ethics Committee of the UCT Faculty of Health Sciences
IFGeneRA: Individual Findings in Genetic Research in Africa
SCD: Sickle cell disease
