# Supplementary material for: Perspectives of researchers, science policy makers and research ethics committee members on the feedback of individual genetic research findings in African genomics research
Source: BMC Med Ethics. 2024 Jun 7;25:67. doi: 10.1186/s12910-024-01068-2 (PMC11157929; doi:10.1186/s12910-024-01068-2)
Supplement: Supplementary file 1 — Supplementary Material 1 [file 12910_2024_1068_MOESM1_ESM.pdf]

Returning Individual Genomic Results Survey

Welcome to the IFGENERA project!

You are being invited to participate in a survey on returning individual genomic research results in Africa.

The purpose of this project is to explore the perspectives of researchers, science policy makers and research ethics committee members on the feedback of individual genetic research findings in genomics research. You are being invited to participate because you fall under one of these groups.

Thank you for agreeing to complete this survey. Your responses are confidential and cannot be linked back to you. We will use the results from this survey to inform recommendations for African genomics research and intend to publish our results. This study was approved by the human health research ethics committee of the UCT Faculty of Health Sciences as part of the IFGENERA study (FHS HREC 782-2018). IFGENERA is part of the H3Africa Consortium. More information can be found on Individual Findings in Genetic Research in Africa (IFGENERA) (h3africa.org). You are not obliged to participate and can decide to stop participating at any time during the survey – in that case, don't submit the responses when prompted. If you agree to participate, you need to complete all questions. The survey should take approximately 10-15 minutes. Data collected through this survey will be kept at UCT for a period of five years after which they will be destroyed. They will be analysed only for the purposes of the proposed study and will not be shared unless necessary for verification or review purposes.

By progressing to the next page, we presume that you give your consent for participation.

By progressing to the next page/ ticking the box below, we will presume that you are happy to participate in this study and give your consent for participation

☐ Yes, I agree

Demographic information

a What is your highest level of education?

◯ Bachelor's degree
◯ Master's degree
◯ Doctoral degree
◯ Other

If other, specify highest level of education:

____

b In which of the following roles have you got most experience with genomics research?
Please tick all that apply

☐ Researcher
☐ Research team leader
☐ Ethics Committee Member
☐ Science Policy Maker
☐ Other

If other, please specify genomics research role:

____

c How long have you been involved in genomics research?

◯ Less than one year
◯ 1-2 years
◯ 3-5 years
◯ 6-10 years
◯ More than 10 years

d How long have you served on the Ethics Committee?

◯ Less than one year
◯ 1-2 years
◯ 3-5 years
◯ 6-10 years
◯ More than 10 years

e How long have you been involved in science policy development?

◯ Less than one year
◯ 1-2 years
◯ 3-5 years
◯ 6-10 years
◯ More than 10 years

Knowledge about Genomics:

1 How confident are you in your understanding of the ethical issues (such as privacy, confidentiality, informed consent and return of individual results) that might arise from genomic research?

◯ Very confident
◯ Somewhat confident
◯ Slightly confident
◯ Not at all confident

2 How familiar are you with issues around the return of individual genetic research results in African genomics (e.g., have you heard about individual findings in training, have you fed back results in the context of genomics research, have you read about individual findings in literature etc.)

◯ Very confident
◯ Somewhat confident
◯ Slightly confident
◯ Not at all confident

3 How familiar are you with the H3Africa Guideline for the Return of Individual Genetic Research Findings?

◯ Very familiar
◯ Somewhat familiar
◯ Slightly familiar
◯ Not at all familiar

Consider adding a comment if you wish to

Views on feedback of individual genetic research findings:

4 Should individual genetic results be returned? Using a scale of 1-5, where 1 suggest strongly agree and 5 disagree how would you rate your view?

Strongly agree Neutral Strongly disagee

(Place a mark on the scale above)

5 Which of the following best characterizes your view on return of individual genetic research results? Please tick all that apply

- ☐ Individual genetic research results should never be fed back in African genomics; there are too many questions and uncertainties
- ☐ Returning some findings is an important way of giving back to the people who participated in the research (reciprocity)
- ☐ Participants have a right to know things about their health that are revealed in African genomics
- ☐ Decisions about what should be returned should be made on a case-by-case basis, and they should be made in consultation with the ethics committee
- ☐ There should be clear guidance about how to go about the return of individual genetic research findings in African genomics
- ☐ Other

If other, specify your view on return of individual genetic research results:

____

What ought to be fed back:

6 When deciding on what information to feedback, what do you think is most important?
Please tick all that apply

- ☐ Participants should have access to as much information as possible
- ☐ Participants should receive all information that is directly relevant to their health
- ☐ Participants should only receive information about their health if that information is clinically actionable ("Clinically actionable" means that there is effective prevention or treatment available through medical care) and could prevent severe disease
- ☐ Participants should receive all information about their health even if that information is not clinically actionable
- ☐ Participants should receive information that could promote a healthier lifestyle and motivate changes in lifestyle
- ☐ Unsure

Consider adding a comment if you wish to

7 Please indicate which of the following best describes your view on returning genetic results to paediatric participants ("Paediatric patients "means persons aged 21 or younger at the time of their diagnosis or treatment). Tick all that apply.

- ☐ Only return results that are clinically actionable in childhood ("Clinically actionable" means that there is effective prevention or treatment available through medical care)
- ☐ Results that are relevant to family members only should be returned
- ☐ Adult-onset conditions should be disclosed ("Adult-onset conditions" means conditions that may manifest themselves after childhood and adolescence.)

8 Genetic findings may be more or less predictive of disease. When deciding to feedback findings, researchers need to decide which kinds of genetic results to feedback. For each of the following options please use a scale from 1 to 5, where 1 represents no obligation to feedback and 5 represents a strong obligation to feedback findings, to indicate your perception of whether researchers have an obligation to feedback genetic research findings. "Clinically actionable" means that there is effective prevention or treatment available through medical care. "Genetic incidental findings (GIFs)" means unexpected findings sometimes called "incidental findings" because the outcome of the test is not related to the initial purpose for which the test was done. Is there an obligation to feedback GIFs that indicates the below listed characteristics?

8.1 Greatly increased risk of a severe disease that is clinically actionable (e.g., pancreatic cancer, breast cancer)

No obligation to disclose — Strong obligation to disclose

(Place a mark on the scale above)

8.2 Greatly increased risk of a severe disease that is not clinically actionable (e.g., Huntington's disease, Alzheimer's disease)

No obligation to disclose — Strong obligation to disclose

(Place a mark on the scale above)

8.3 Greatly increased risk of a mild or moderate disease that is clinically actionable (e.g., arthritis, hypertension, gout)

No obligation to disclose — Strong obligation to disclose

(Place a mark on the scale above)

8.4 Greatly increased risk of a mild or moderate disease that is not clinically actionable (e.g., chronic fatigue, dyslexia)

No obligation to disclose | Strong obligation to disclose

(Place a mark on the scale above)

8.5 No disease risk, but IF is relevant for reproductive decision-making (e.g., carrier status for heritable conditions such as SCD)

No obligation to disclose | Strong obligation to disclose

(Place a mark on the scale above)

8.6 Greatly increased chance of familial kidney disease

No obligation to disclose | Strong obligation to disclose

(Place a mark on the scale above)

8.7 Pharmacogenomic information that indicates which medications/drugs will work more effectively for that individual

No obligation to disclose | Strong obligation to disclose

(Place a mark on the scale above)

8.8 Greatly increased chance of displaying non-medical traits, such as the chance of going bald

No obligation to disclose | Strong obligation to disclose

(Place a mark on the scale above)

Consent:

9 At which point should participants give consent to receive incidental findings?

◯ Only at the time of enrolment
◯ When relevant findings are identified that should be fed back
◯ Both at the time of enrolment and again when relevant findings are identified
◯ At another time, namely
◯ Unsure

If at another time, specify

____

10 A participant has chosen on the consent form not to receive any IF results. During analysis, the research team finds evidence of high genetic risk for a hereditary form of cancer that is unlikely to have been diagnosed. The team believes this information will prevent serious disease and could save the life of the participant. Should the team disclose the finding, even though the participant indicated that he/she did not want to receive any IFs?

◯ Yes, they should definitely disclose the finding
◯ Yes, they should probably disclose the finding
◯ No, they should probably not disclose the finding
◯ No, they should definitely not disclose the finding
◯ Unsure

Actionability:

11 Generally, the consensus is that findings that are actionable - i.e. where some treatment or behaviour intervention is available to prevent the condition - should be fed back. The IFGENERA research participants have indicated that they would like to receive as many findings as possible. In some situations, we are concerned that even if findings are technically clinically actionable, participants may not in fact have access to the intervention that would prevent the condition in low-income settings. An example is identifying a genetic risk for breast cancer for which mastectomy (surgical removal of the breasts) would be recommended. This surgery may not in fact be available to research participants in settings with poor healthcare infrastructure. In that case, would you say:

◯ Participants should still receive findings in the hope that they would be able to access surgery somewhere, somehow
◯ Participants should not receive findings for which they are unlikely to be able to access treatment or prevention
◯ Ask each participant if they would like to receive this kind of finding
◯ Unsure

Standard of care:

12 Most African genomics research takes place across multiple countries, each of which may have different standard of care. Also, some projects may collect samples in rural areas and others may collect samples in urban areas (where there may be better healthcare available). Considering this, what should researchers consider when deciding which results to feedback?

◯ The local standard of care (i.e. what care participants can access locally)
◯ The national standard of care in the country where the research takes place
◯ The international best practice/standard of care should apply
◯ Unsure

13 Where projects work across different settings in Africa, should the same or different standard of care apply?

◯ The same standard of care should be applied across the entire project
◯ Local or regional availability of treatment should guide a decision about what results are returned rather than one standard should apply across the project
◯ International standards of care should apply
◯ Unsure

Reproductive decision making:

14 Sickle cell disease (SCD) is a severe hereditary form of anaemia in which a mutated form of haemoglobin distorts the red blood cells into a crescent shape at low oxygen levels). If both parents have the gene, there's a 1 in 4 chance of each child they have being born with sickle cell disease. In your view, is it appropriate to return information about SCD carrier status in countries with a high burden of SCD?

◯ Yes
◯ No
◯ Unsure

Constraints in returning incidental findings:

15 In your view, what are the most important constraints in returning Incidental Findings in African genomics research? Please tick all that apply

- ☐ Capacity to analyse large datasets for Incidental Findings
- ☐ Capacity to interpret the relevance of findings for the health of individuals
- ☐ Absence of genetic health professionals (trained genetic nurses, genetic counsellors and medical geneticists)
- ☐ Difficulty of establishing pathogenicity of variants due to poor representation of Africans in genetic databases
- ☐ Cost of returning results
- ☐ Absence of genetic diagnostic testing facilities to confirm research findings
- ☐ Other

If other, specify constraints in returning Incidental Findings:

____

Importance of feeding back findings:

16 What is your view on the importance of feedback of individual genetic research results?
Please tick all that applies

- ☐ Return of research results would be one way in which research participation could be reciprocated
- ☐ Feedback of individual genetic research results could be a way of appreciating participants' contribution to research
- ☐ Participants should receive their individual genetic research results in exchange for their participation in research
- ☐ Receiving individual genetic research results would show participants that their participation was valued

Cost of feeding back findings:

17 Who should incur the costs of feeding back results?

◯ Project Funders
◯ Institution hosting research
◯ Researchers
◯ Participants
◯ Other

If other, specify Who should incur the costs of feeding back result:

____

Experience on return of findings:

18 Please share with us relevant information about your experience or views on return of findings that we did not capture in this survey/ that you would still like to share with us:
